# Continuous Metabolic Syndrome Scores for Children Using Salivary Biomarkers

**DOI:** 10.1371/journal.pone.0138979

**Published:** 2015-09-29

**Authors:** Ping Shi, J. Max Goodson, Mor-Li Hartman, Hatice Hasturk, Tina Yaskell, Jorel Vargas, Maryann Cugini, Roula Barake, Osama Alsmadi, Sabiha Al-Mutawa, Jitendra Ariga, Pramod Soparkar, Jawad Behbehani, Kazem Behbehani, Francine Welty

**Affiliations:** 1 Department of Applied Oral Sciences, the Forsyth Research Institute, Cambridge, Massachusetts, United States of America; 2 The Dasman Diabetes Institute, Kuwait City, Kuwait; 3 Ministry of Health, Kuwait City, Kuwait; 4 Faculty of Dentistry, Kuwait University, Kuwait City, Kuwait; 5 Division of Cardiology, Beth Israel Deaconess Medical Center, Boston, Massachusetts, United States of America; University, ITALY

## Abstract

**Background:**

Binary definitions of the metabolic syndrome based on the presence of a particular number of individual risk factors are limited, particularly in the pediatric population. To address this limitation, we aimed at constructing composite and continuous metabolic syndrome scores (cmetS) to represent an overall measure of metabolic syndrome (MetS) in a large cohort of metabolically at-risk children, focusing on the use of the usual clinical parameters (waist circumference (WC) and systolic blood pressure (SBP), supplemented with two salivary surrogate variables (glucose and high density lipoprotein cholesterol (HDLC). Two different approaches used to create the scores were evaluated in comparison.

**Methods:**

Data from 8,112 Kuwaiti children (10.00 ± 0.67 years) were used to construct two cmetS for each subject. The first cmetS (cmetS-Z) was created by summing standardized residuals of each variable regressed on age and gender; and the second cmetS (cmetS-PCA) was defined as the first principal component from gender-specific principal component analysis based on the four variables.

**Results:**

There was a graded relationship between both scores and the number of adverse risk factors. The areas under the curve using cmetS-Z and cmetS-PCA as predictors for severe metabolic syndrome (defined as the presence of ≥3 metabolic risk factors) were 0.935 and 0.912, respectively. cmetS-Z was positively associated with WC, SBP, and glucose, but inversely associated with HDLC. Except for the lack of association with glucose, cmetS-PCA was similar to cmetS-Z in boys, but had minimum loading on HDLC in girls. Analysis using quantile regression showed an inverse association of fitness level with cmetS-PCA (*p* = 0.001 for boys; *p* = 0.002 for girls), and comparison of cmetS-Z and cmetS-PCA suggested that WC and SBP were main contributory components. Significant alterations in the relationship between cmetS and salivary adipocytokines were demonstrated in overweight and obese children as compared to underweight and normal-weight children.

**Conclusion:**

We have derived continuous summary scores for MetS from a large-scale pediatric study using two different approaches, incorporating salivary measures as surrogate for plasma measures. The derived scores were viable expressions of metabolic risk, and can be utilized to study the relationships of MetS with various aspects of the metabolic disease process.

## Background

Metabolic syndrome (MetS) has been defined as a concurrence of metabolic abnormalities associated with atherosclerotic cardiovascular disease and insulin resistance that are related to the development of type 2 diabetes (T2D). Based on recommendations from the International Diabetes Federation (IDF) and Adult Treatment Panel III (ATPIII) [1], the core components of MetS include elevated abdominal adiposity, blood pressure, glucose, and triglycerides, and lowered high-density lipoprotein cholesterol, considered present if exceeding certain threshold values. MetS is well defined in the adult population, but due to relatively low prevalence rate (<10%) [2, 3] and lack of large-scale studies, its definition is not as clear in children and adolescents, especially in terms of choice of factors to be included at this early stage when some symptoms have yet to emerge.

Despite the usefulness of a binary definition of individual risk factor in clinical setting, by which diagnosis of the metabolic syndrome is made in the presence of a certain number of measures exceeding thresholds (e.g. n ≥ 3), growing evidence supports using a continuous approach instead of a dichotomous one [4–8]. Aside from the statistical consideration to gain power by using all the information, it is believed that the risk of MetS increases gradually with increasing levels of each individual risk factor, together with increasing number of risk factors. Therefore, a continuous score would best reflect the progressive nature by providing a measure in risk severity. Ideally, an approach would allow for differential weighting of different components [9, 10], accounting for the fact that not all risk factors contribute equally to MetS, which is an assumption inherent in the dichotomous approach.

Various strategies have been used to construct a continuous metabolic syndrome scores (cmetS) from its components. The most frequently used methods are the summation of standardized Z scores adjusting for covariates [7, 11], and the use of first principal component (PC1) from principal component analysis (PCA) [12–15]. Z score summation is an efficient method, but it is limited by the presumption that each component contributes equally and independently to the total risk, thereby assigning the same weight to each measure and failing to address the inter-correlation among them. PCA, on the other hand, is an analytical approach that has been designed to summarize multidimensional correlated data. Its unrotated PC1 is the linear combination of all measures that captures the maximum variance in the data, more than any other linear combination from succeeding PCs. Thus, PC1 is a reasonable representation for quantifying MetS, by assigning differential weights to different components with variable loadings, and accounting for the maximum variance among the components. Compared to the Z score approach, PCA is more data-driven in the sense that it allows the data to determine how much each individual risk component contributes to the process, instead of imposing that all components contribute equally. This is especially helpful in the case of pediatric studies, where there is no universally accepted definition of MetS and the relative importance of various components is less certain than it is in adults.

The current analysis is based on a large-scale longitudinal study evaluating factors related to the etiology and development of obesity in over 8,000 Kuwait children aged 8–14 years [16]. In this population, obesity and hypertension were prevalent (34.2% and 23.9%, respectively), and the prevalence of metabolic syndrome (presence of at least 3 risk factors) was approximately 1.1%. Given the fact that we have a relatively large pediatric population with a low prevalence of MetS from a region where adult T2D is highly prevalent [17], it is of particular interest to derive a continuous score representing a composite risk profile of MetS still at its emergent stage, in which the interplay of different components may be quite different from adults, and metabolic risk is difficult to assess as defined by the binary variable. The cmetS score will be tracked in the follow-up study as an indicator of metabolic risk, and cmetS at baseline can be used in predicting later incidents, such as development of T2D or cardiovascular disease.

Importantly, one aspect worth noting with this cohort is that fasting saliva samples were collected in all subjects and risk factors and many biomarkers were measured, making it possible to evaluate MetS profile using saliva parameters as surrogates of plasma parameters.

Based on the unique metabolic risk status of this study population and the increasing utility of cmetS in pediatric research, the focus of our study is to construct and validate cmetS using two different approaches: Z score and PCA. Furthermore, the utility of the scores was evaluated in terms of predicting fitness level, as well as examining the relationship between salivary adipocytokines and metabolic risk.

## Materials and Methods

This research study was part of the Kuwait Healthy Life Study (KHLS), which aimed to investigate the obesity-related consequences and the etiology of metabolic syndrome in Kuwait children. The validation study for US subjects was approved by the Forsyth Institutional Review Board in USA, and the study of Kuwaiti subjects (4th or 5th grades) was approved by the Dasman Diabetes Institute Ethical Review Committee in Kuwait. Written consent forms signed by parents/guardians were collected in advance for both US and Kuwait study subjects. Subject assent was obtained on the day of the visit.

Saliva samples were collected rather than blood samples in recognition of salivary assay as a source of valuable surrogate information [18, 19], to reduce anxiety in our Kuwait subjects (10 year old children) [20] and to promote their willingness to participate in our study. The use of non-invasive procedures throughout the study was directly related to our ability to enroll over 8,000 children/year within the Kuwaiti school system

### Study population

KHLS has been previously described [16]. Briefly, clinical data and saliva samples were collected from 8,319 participants during 179 visits to 138 Kuwaiti schools between October 2, 2011 and May 15, 2012. Participating children were 4th and 5th graders attending Kuwaiti public schools, which were approximately equally distributed among each region (governorate) of Kuwait. After excluding those with missing saliva samples and major clinical measurements, 8,112 children were included in the current study, predominantly pre-pubertal based on the respective age criteria for boys and girls. A few missing values for clinical covariates were imputed by median values.

### Clinical and biological measurements

Subject identification, height, weight, blood pressure, oral examination findings, and fitness data were entered into a programmed iPad™ (Apple, Cupertino, CA) system for Internet transfer, as described previously [16]. Body weight categories were defined by World Health Organization (WHO) criteria using a Body Mass Index (BMI) Z-score [21]. Based on this criterion, obese was ≥95^th^ percentile, overweight was ≥85^th^ to <95^th^ percentile, normal healthy weight was ≥5^th^ to <85^th^ percentile and underweight was <5^th^ percentile. Fitness was measured by heart rate elevation (beats/minute) following a standard 3-minute exercise [22]. Another obesity status was defined according to the criteria based on the waist circumference (WC). Based on the data from European-American children and adolescents [23], those with ≥90^th^ percentile in WC for age and sex were assigned as obese.

Saliva samples were collected after overnight fast, as described previously [24]. Samples were centrifuged at 2,800 rpm for 20 min at 4°C, after which the supernatants were transferred to a screw-cap 2D barcoded storage tubes (Thermo Scientific), which can be read by a barcode reader (Thermo Scientific VisionMate™ ST) and frozen at -80°C. The frozen samples were air-transported to Forsyth Institute under temperature-monitored dry ice.

High-density lipoprotein cholesterol (HDLC) and glucose were measured in all the saliva samples, using fluorescent spectroscopic analysis (Infinate® 200 Pro, Tecan, Gröndig, Austria) from commercially available kits (BioVision, Mountain View CA, HDL and LDL/VLDD Cholesterol Quantification Kit, #K613-100 and Glucose Assay kit Cat #K606-100) adapted to operate on a robotic chemical assay platform (Tecan EVO 150,Tecan Group, Männedorf, Switzerland).

We randomly selected 744 samples to assay for 20 biomarkers (insulin, CRP, adiponection, leptin, IL-1β, IL-4, IL-6, IL-8, IL-10, IL-12P70, IL-13, IL-17A, resistin, MMP_9, MPO, MCP-1, TNF-α, VEGF, IFN-γ, and ghrelin), as previously described [24]. Briefly, saliva supernatants were thawed at 4°C overnight and kept on ice throughout the assay procedure. All assays were performed on 25 μl of saliva supernatant using four multiplex magnetic bead panels on a Luminex 200™ system (Luminex, Austin, TX). Results were evaluated using Bio-Plex Manager™ (Version 5.0; Bio-Rad, Hercules, CA). IL-17A, IFN-γ, and ghrelin were not included in analysis due to a large number of zero or abnormal values. Additionally, 18 samples with extreme values in measurements were excluded from the current analysis, as assessed by their undue influence on regression in an initial model.

### Statistical analyses

All of the statistical analyses were performed using SAS version 9.3 (SAS Institute, Cary, NC, USA). The risk factor measures chosen to construct both metabolic syndrome scores (cmetS-Z and cmetS-PCA) were: waist circumference (WC), systolic blood pressure (SBP), salivary HDLC concentration, and salivary glucose concentration.

#### Construction of cmetS-Z

The derivation of the cmetS-Z involved two steps. First, for each risk factor, standardized residuals (Z score) were calculated by regressing them onto age and gender to adjust for age and gender differences. Z scores for HDLC were multiplied by –1, based on the assumption that HDLC is inversely associated with MetS risk; Secondly, Z scores for each measure were summed to create a continuous composite score (cmetS-Z = Z_WC + Z_SBP—Z_Saliva HDLC + Z_saliva glucose) This approach resulted in each risk component being equally weighted in the final score.

#### Construction of cmetS-PCA

We constructed cmetS-PCA using principal component analysis (PCA). PCA transformed the original variables into a set of principal components (PC), with the first PC (PC1) being the linear combination of all variables that captured the largest variance in the data, and each succeeding PC as the one that captured the largest fraction of variance orthogonal to the preceding PC. On the log-transformed measures of the four risk factors, PCA was performed using the correlation matrix of standardized variables, stratified by gender. As PC1 accounts for the largest proportion of total variance in the four measures, we defined it as a continuous score of metabolic syndrome (cmetS-PCA). In contrast to the Z score method, this approach allowed for differential weighting of each individual risk component in the final score.

#### Analysis using cmetS

MetS was defined as the presence of at least three of the following four factors: 1) WC≥90^th^; 2) SBP≥130 or DBP≥85; 3) salivary HDLC≤0.6 mg/dL (approximate plasma HDLC ≤50 mg/dL) (S1 Appendix); and 4) salivary glucose ≥1.13 mg/dL (approximate plasma level of glucose ≥ 100mg/dL) [25]

The ability of cmetS to predict MetS was assessed using receiver operating characteristic (ROC) curves. The area under the curve (AUC) was taken as a measure of overall accuracy of cmetS to discriminate between subjects with and without severe metabolic syndrome.

Quantile regression (SAS 9.3, Proc Quantreg) was used to assess the association of fitness with cmetS, stratified by gender and adjusting for potential confounders including age, BMI, sleep parameters, and regions in which the patient’s school was located. This technique was used to estimate conditional quantiles, requiring no distribution assumption of the dependent variable and was robust to outliers [26]. The linear quantile regression function Q (*τ*|*X* = *x*) = *x*′*β*(*τ*) can be estimated by solving $\hat{\beta }(\tau )=argmin\sum_{i=1}^{n}\rho_{\tau }(y_{i}-x_{i}^{\prime }\beta )$, where *ρ*_*τ*_(*u*) = *u*(*τ* − *I*_(*u*<0)_), for any quantile *τ* in [0,1]. We chose *τ* = 0.5 thus it corresponded to median regression. As fitness had an extremely skewed distribution, quantile regression offered a robust alternative to linear regression for modeling this variable.

In the analysis of the random cohort in which 20 salivary biomarkers were assayed, model selection based on stepwise selection was used to develop a parsimonious linear model to predict cmetS (Sas 9.3, Proc Glmselect). As the distribution of both cmetS scores was approximately normal, they could be reasonably modeled by a linear model. During the selection process, all biomarkers (values standardized) and variables of subject features were considered. The Schwartz Bayesian Criterion was evaluated for all models by deleting a variable from the current model or adding a variable to this model. To avoid over-fitting, a 10-fold cross validation procedure was adopted to assess the performance, and the best model was determined based on the average predictive performance in the test sets. Subsequently, the selected subsets of significant predictors for each cmetS were examined separately in subgroups, stratified by their WHO body weight categories.

## Results

### Anthropometric and metabolic phenotypes of participants

In the KHLS study, 3045 boys and 5067 girls (total n = 8112) completed clinical measures of BMI, waist circumference, and blood pressure, as well as HDLC and glucose levels in fasting saliva samples collected at baseline (Table 1). Boys appeared to have higher metabolic risk than girls in regard to blood pressure measures (*p*<0.0001 for SBP, *p* = 0.0002 for DBP), salivary glucose level (*p*<0.0001), and the proportion identified as obese (*p*<0.0001). Girls, however, had a significantly lower salivary HDLC level than boys (*p*<0.0001)

**Table 1:** Characteristics of study participants (mean (SD)).

Characteristic	Boys (n = 3045)	Girls (n = 5067)	*p* value	Combined (n = 8112)
Age (years)	9.99 (0.67)	10.00 (0.67)	0.78	10.00 (0.67)
Weight (kg)	40.38 (13.75)	40.26 (12.87)	0.67	40.31 (13.21)
Height (cm)	137.3 (7.56)	137.8 (7.69)	0.004	137.7 (7.64)
BMI (kg/m^2^) ^**1**^	21.01 (5.34)	20.86 (5.14)	0.26	20.92 (5.22)
% obese (WHO category) ^**2**^	39.21%	31.24%	<0.0001	34.23%
Waist circumference (cm)	68.22 (12.79)	68.43 (11.80)	0.46	68.35 (12.18)
Systolic blood pressure (mmHg)	110.4 (17.64)	108.9 (15.39)	<0.0001	109.44 (16.29)
Diastolic blood pressure (mmHg)	74.43 (13.36)	73.30 (12.44)	0.0002	73.72 (12.81)
Saliva HDLC (mg/dL) ^**1**^	1.36 (1.06)	1.26 (1.02)	<0.0001	1.30 (1.04)
Saliva glucose (mg/dL) ^**1**^	0.22 (0.28)	0.18 (0.22)	<0.0001	0.19 (0.24)
Fitness level (Δbpm) ^**1**^	25.31 (22.45)	26.34 (22.53)	0.02	25.96 (22.50)
Metabolic syndrome^**2**^	1.22%	1.11%	0.65	1.14%

Comparison between sexes was by t-test, except as noted.

^**1**^ via Wilcoxon rank test.

^**2**^ via Chi-square test.

### Construction of cmetS-Z and cmetS-PCA and their validity as indicators of MetS

The value of cmetS-Z for each child was obtained by summing the standardized residual for individual components of risk factors (i.e. WC, SBP, Saliva HDLC and glucose), after regressing onto age and gender. Meanwhile, PCA was performed using the log-transformed components, stratified by gender. In boys, the first PC (PC1) explained 37.2% of the variance (Eigenvalue = 1.49). The loadings of the four components were: WC: 0.70, SBP: 0.70, HDLC: -0.13, glucose: -0.04. In girls, PC1 explained 35.9% of variance (Eigenvalue = 1.43), and the loadings of the four components were: WC: 0.70, SBP: 0.71, HDLC: 0.02, glucose: -0.03. Notably, HDLC had a negative loading in boys, consistent with the notion that HDLC is inversely related to metabolic risk. In girls, however, the loading of HDLC was close to zero. Because of this difference in the loading profile, the two groups of participants were not combined for subsequent analysis. To ensure that the score was maximally spread out the four components, cmetS-PCA was constructed using an unrotated PC1.

Table 2 shows the graded relationship between each cmetS score and number of risk factors. We found that cmetS-Z was the lowest in children with no risk factors (-1.11±1.62) and highest (4.46±2.71) in the group with 3 risk factors. Likewise, cmetS-PCA increased progressively with number of risk factors from no risk (-0.63±0.87), to 1 risk factor (0.46±0.99), 2 risk factors (1.62±0.87), and 3 risk factors (1.86±0.65). It is worth noting that as number of risk factors increased, in contrast to the significant changes in the values for WC and SBP, values for salivary levels of HDLC and glucose did not exhibit substantial change until the number reached 3. The lack of change in these two components in the study population was reflected in PC1, which had a small negative loading on HDLC only in boys and nearly zero loading on glucose in both sexes.

**Table 2:** Values of continuous metabolic syndrome scores stratified by number of risk factors (mean (SD)).

Number of risk factors* present	Waist circumference (cm)	Systolic blood pressure (mmHg)	Saliva HDLC (mg/DL)	Saliva Glucose (mg/DL)	cmetS-Z	cmetS-PCA	Age (years)	N
0	62.46 (7.00)	102.5 (12.5)	1.39(1.07)	0.18 (0.16)	-1.11 (1.62)	-0.63 (0.87)	9.99 (0.67)	4550
1	72.15 (12.20)	114.8 (15.7)	1.20(0.99)	0.22 (0.30)	0.84 (1.75)	0.46 (0.99)	9.99 (0.65)	2519
2	84.65 (11.20)	126.6 (14.7)	1.18 (0.94)	0.22 (0.35)	2.64 (1.86)	1.62 (0.87)	10.04 (0.70)	950
3	86.86 (8.90)	129.0 (11.9)	0.70 (0.47)	0.47 (0.64)	4.46 (2.71)	1.86 (0.65)	10.04 (0.64)	92
4	88.90	120.0	0.48	1.18	7.13	1.64	10.01	1

* Risk factors include obesity defined by abdominal adiposity, blood pressure, fasting saliva HDLC and glucose concentrations expressed in calibrated serum concentrations, with values surpassing a threshold value as described in Materials and Methods.

**Fig 1:**
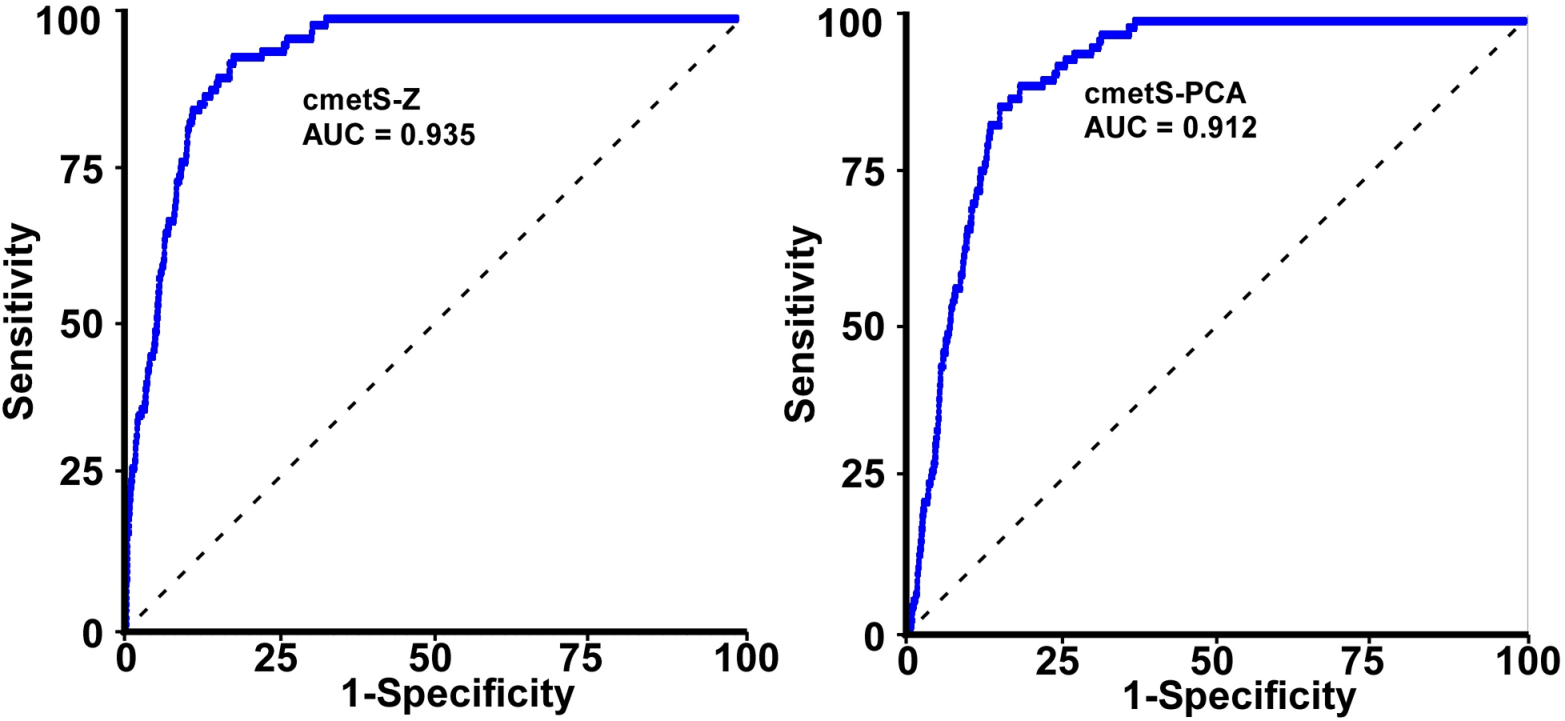
ROC for cmetS-Z and cmetS-PCA as predictors for MetS in Kuwait children.

To evaluate the global accuracy of cmetS-Z and cmetS-PCA as predictors for MetS (defined as presence of > = 3 risk factors), ROC curve analysis was performed (Fig 1). The AUC for cmetS-Z was 0.935, and 0.912 for cmetS-PCA. Both scores had AUCs that outperformed the AUCs of individual metabolic risk components as follows: WC (0.892), SBP (0.844), salivary HDLC (0.851), and salivary glucose (0.593) (Table 3). It was found that PC2, whose loadings were largely on HDLC and glucose (boys: WC: 0.03, SBP: 0.14, HDLC: 0.67, glucose: 0.73; girls: WC: 0.07, SBP: -0.05, HDLC: 0.62, glucose: 0.78), was not a good predictor of MetS (AUC = 0.602). Based on the fact that PC2 did not constitute viable expressions of metabolic risk, PC1 alone was chosen to construct cmetS-PCA, instead of using the combined vectors of PC1 and PC2, which would have attenuated the predictive power of PC1.

**Table 3:** Area under the curve (AUC) of cmetS-Z and cmetS-PCA as predictors for MetS (defined as the presence of > = 3 risk factors), as compared to that using the individual components as predictors. AUCs using these predictors to predict an emerging state of MetS (defined as the presence of > = 2 risk factors) are also presented.

	cmetS-Z	cmetS-PCA	Waist circumference (cm)	Systolic blood pressure (mmHg)	Saliva HDLC (mg/DL)	Saliva Glucose (mg/DL)
AUC (Risk factor > = 3)	0.935	0.912	0.892	0.844	0.851	0.593
AUC (Risk factor > = 2)	0.900	0.917	0.894	0.841	0.593	0.513

Despite the low prevalence of MetS in this study population (1.15%), there was a much larger group with at least 2 risk factors present (12.86%). Such children may be susceptible to developing MetS later in life. Therefore, we also tested an alternative outcome among children using a less stringent criterion for MetS (i.e. ≥2 risk factors present). As shown in Table 3, we found that cmets-PCA had the highest AUC (0.917), followed by cmetS-Z (0.900), WC alone (0.894) or SBP alone (0.841). Notably, although salivary HDLC level was a good predictor for MetS, it was not a good predictor (0.593) for this emerging state of MetS.

**Fig 2:**
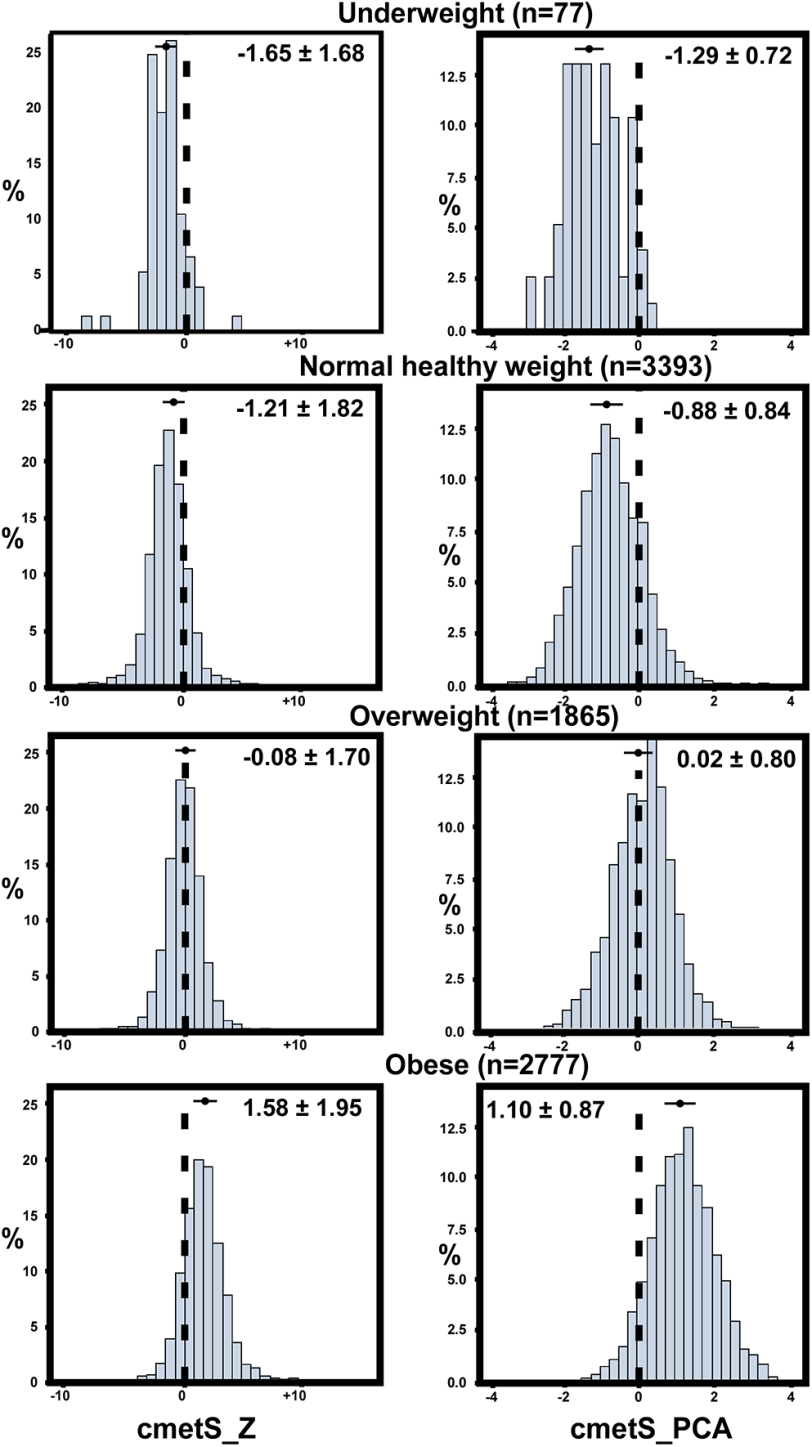
Distribution of cmetS-Z and cmetS-PCA across WHO body weight categories in a cohort of 8,112 Kuwaiti children. The four body weight categories were defined according to WHO criteria. The numbers at the top of each graph were the mean ± SD of cmetS-Z and cmetS-PCA for each category, as also indicated by the bars at the corresponding locations of the distributions.

To examine the metabolic risk in obese children as compared with healthy-weight children, average cmetS-Z and cmetS-PCA were compared across WHO body weight categories. Both obese and overweight children had significantly higher scores than did normal-weight children (cmetS-Z: 1.58±1.95 and -0.08±1.70 vs. –1.21±1.82; cmetS-PCA: 1.10±0.87 and 0.02±0.80 vs. -0.88±0.84), indicating a significant risk even existed in overweight children. Correspondingly, the distribution of these two scores exhibited a gradual shift towards higher values across the four body weight categories (Fig 2)

### Association of cmets-Z and cmets-PCA with fitness level

The association of both cmetS scores with fitness was assessed using quantile regression. The analysis was stratified by gender, adjusting for age, BMI, sleep parameters, and region (Table 4). In both boys and girls, cmetS-PCA, but not cmetS-Z, was found to be significantly associated with fitness level, conditioned on the above covariates. In boys, for every 1 SD increase in cmetS-PCA there was an average increase of 3.24 beats/min in heart rate (*p* = 0.001). In girls, for every 1 SD increase in cmetS-PCA there was an average increase of 2.01 beats/min in heart rate (*p* = 0.002). Thus, cmetS-PCA demonstrated a strong association with fitness level in children, but not cmets-Z, although it exhibited a marginal significant association with fitness level in girls (*p* = 0.07).

**Table 4:** Association of fitness level with cmetS-Z and cmetS-PCA, adjusting for age, sex, BMI, sleep parameters, and region in which the participants’ school was located.

	Fitness vs. cmetS-Z		Fitness vs. cmetS-PCA	
	Estimate (95% CI)	*p* value	Estimate (95% CI)	*p* value
**Boys (n = 3045)**				
Age (per year)	1.62 (0.04–3.19)	0.04	1.14 (-0.39–2.68)	0.14
cmetS (per SD)	1.02 (-0.44–2.48)	0.17	3.24 (1.27–5.22)	0.001
BMI(per unit)	0.82 (0.55–1.08)	<0.0001	0.40 (0.03–0.77)	0.03
Sleep (per hr)	0.40 (-0.25–1.05)	0.23	0.33 (-0.31–0.99)	0.31
Region		0.01*		0.02*
**Girls (n = 5067)**				
Age (per year)	-0.43 (-1.56–0.69)	0.45	-0.62 (-1.74–0.50)	0.28
cmetS (per SD)	0.84 (-0.07–1.76)	0.07	2.01(0.77–3.25)	0.002
BMI(per unit)	0.85 (0.68–1.01)	<0.0001	0.67 (0.45–0.90)	<0.0001
Sleep (per hr)	0.33(-0.12–0.78)	0.15	0.38 (-0.08–0.85)	0.11
Region		<0.0001*		0.0001*

*indicates *p* values of Wald test for all categories in type 3 analysis.

### Salivary biomarkers as predictors of cmetS-Z and cmetS-PCA

In a previously reported randomly selected cohort of 744 children [24], various salivary biomarkers including adipocytokines were assayed. In the current analysis, all biomarkers and variables of subject characteristics were considered for building a predictive model for metabolic risk as indicated by cmetS. Data from the 726 subjects of the entire cohort was used for stepwise selection, for the benefit of large sample size. This selection procedure with 10-fold cross validation yielded a parsimonious model for each score, which, for cmetS-Z, consisted of CRP, insulin, adiponectin, and fitness status. For cmetS-PCA, the procedure yielded an almost identical subset, except leptin was added as an additional predictor. Further analysis was conducted in two sub-groups divided by WHO body weight category: normal weight and underweight versus overweight and obese. In each sub-group, a predictive model for cmetS-Z or cmetS-PCA was built based on the above identified predictors. As shown in Table 5, in the normal weight/underweight sub-group, neither biomarkers nor fitness status was a significant predictor for cmetS scores, The one exception was adiponectin, which was inversely associated with cmetS-Z (*p* = 0.01). In contrast, in the overweight/obese sub-group most biomarkers became significant predictors for cmetS, with insulin being the strongest predictor for both cmetS-Z (*p*<0.0001) and cmetS-PCA (*p*<0.0001), followed by adiponectin (*p* = 0.0002 and *p* = 0.007, respectively), CRP (*p* = 0.01 and *p*<0.0001, respectively), and fitness status (*p* = 0.07 and *p* = 0.003, respectively). Leptin, although was selected as a significant predictor for cmetS-PCA from the entire cohort, did not manifest as such in the overweight/obese sub-group.

**Table 5:** Multiple salivary biomarkers and subject characteristics as predictors for cmetS-Z and cmetS-PCA in a randomly selected cohort (n = 726), stratified by WHO obesity status

	cmetS-Z		cmetS-PCA	
	Estimate (95% CI)	*p* value	Estimate (95% CI)	*p* value
**Underweight and normal-weight children (n = 312)**				
Age (per year)	0.15 (-0.15–0.45)	0.33	0.30 (0.15–0.45)	0.0001
CRP (per SD)	-0.04 (-0.23–0.16)	0.72	0.009 (-0.09–0.11)	0.86
Insulin (per SD)	0.11 (-0.35–0.59)	0.64	0.13 (-0.11–0.37)	0.29
Adiponectin (per SD)	-0.21 (-0.37–-0.05)	0.01	-0.02 (-0.11–0.06)	0.57
Leptin (per SD)	NA	NA	-0.03 (-0.15–0.23)	0.62
Fitness (poor vs. good)*	0.02 (-0.35–0.40)	0.90	0.04 (-0.15–0.23)	0.71
**Overweight and obese children (n = 414)**				
Age (per year)	0.28 (-0.03–0.59)	0.08	0.36 (0.23–0.49)	<0.0001
CRP (per SD)	0.28 (0.07–0.48)	0.01	0.18 (0.10–0.27)	<0.0001
Insulin (per SD)	0.48 (0.32–0.65)	<0.0001	0.19 (0.12–0.26)	<0.0001
Adiponectin (per SD)	-0.47 (-0.71–-0.22)	0.0002	-0.14 (-0.25–-0.04)	0.007
Leptin (per SD)	NA	NA	0.04 (-0.03–0.12)	0.28
Fitness (poor vs. good)	0.36 (-0.04–0.76)	0.07	0.25 (0.09–0.42)	0.003

*poor is defined as having values above the median, and good as having values below the median.

## Discussion

In this population of 8,112 children who are at risk of metabolic disease, we used two approaches to construct continuous metabolic syndrome scores (cmetS) in order to summarize the inter-correlated risk factors for MetS. The first approach was based on the sum of sample-specific Z scores (cmetS-Z), and the second was based on the first principal component from principal components analysis (cmetS-PCA). We assessed their respective global accuracy in predicting MetS and compared each to that of individual metabolic risk components. We also evaluated the predictive utility of these scores by their ability to predict cardiovascular health as measured by fitness level, and investigated their relationships with salivary adipocytokines in subgroups within different body weight categories

Since it is not clear whether the definition of metabolic risk of adulthood can be extended to childhood where MetS is at its emergent stage, there has been little consensus on the criteria for defining MetS in children and adolescents, both in terms of the choice of risk factors and their respective threshold values [27]. In this context, it is useful to develop a continuous summary score that takes into account of all risk components and provides a progressive measure for the severity of metabolic risk, either in equally weighted [7] or differentially weighted manner [28].The two summary measures developed within a large and homogeneous pediatric population in this study enabled us to compare these two strategies.

In our study, variables of risk factors used to derive cmetS were selected *a priori*, which included waist circumference (WC), systolic blood pressure (SBP), saliva HDLC, and saliva glucose. As triglycerides and HDLC have a strong inverse correlation [29, 30] we chose saliva HDLC to represent the lipid parameters due to measurement issues of salivary triglycerides in our study. For the Z score method, these four variables were pre-defined as contributors to metabolic risk, each being presumed equally important. In contrast, PCA, as a method that transforms correlated variables to a set of uncorrelated principal components (PC), had differential loading coefficient for each variable in the PCs. Algebraically, the first PC captured the maximum variance in the data, so it follows that PC1 was the quantity that most efficiently characterized the clustering of original variables, and for that reason it was defined as the index score representing metabolic risk. Interestingly, PC1 was found to be considerably loaded on WC and SBP in both boys and girls, marginally loaded on saliva HDLC in boys, and not loaded on saliva glucose in either. Correspondingly, prominent changes of WC and SBP were observed across different risk factor categories, which contrasted with relatively small changes seen in salivary HDLC level and marginal changes in saliva glucose level across categories, especially the first three in which most subjects belonged to (Table 2). This suggests that the main drivers of metabolic risk in this population are WC and SBP, together with a small contribution from HDLC. Hence, unlike the summed Z score assuming equal weighting of each component, the data-driven PCA approach created a summary measure of differential weighting that minimized the role of glucose in this population, which was consistent with earlier observations that fasting blood glucose was typically normal in youth and even in overweight youth [31].

The two cmetS constructed in our study increased progressively with increasing number of risk factors (Table 2). As shown by ROC analysis, cmetS-Z and cmetS-PCA performed well in predicting MetS as conventionally defined in a clinical setting, (AUC = 0.935, 0.912, respectively), and was superior to that using individual component (Table 3). We also tested an alternative outcome using a less stringent criterion for MetS (i.e., ≥2 risk factors), and found cmetS-Z and cmetS-PCA are also good predictor for this outcome (AUC = 0.900, 0.917, respectively). In this scenario, HDLC was not a predictor for the outcome anymore, suggesting that in the early stage of MetS, WC and SBP were the main emergent components. As an obesity-related consequence, metabolic risk increased along with obesity status, as demonstrated in Fig 2, in which distributions of both scores showed a marked upward shift across WHO body weight categories (Fig 2).

Previous studies suggested that physical fitness, especially cardiorespiratory or aerobic fitness, is correlated with metabolic risk [32, 33]. Thus we used fitness level as an outcome that may have long-term implication for cardiovascular functions. In the analysis stratified by gender, fitness level was found to be highly significantly associated with cmetS-PCA (*p* = 0.001 for boys, 0.002 for girls), independent from BMI which was always a strong predictor of fitness (Table 4). In the case of cmetS-Z, however, a marginally significant relationship with fitness was only found in girls (*p* = 0.07). The fact that cmetS-PCA demonstrated a stronger association with fitness than cmetS-Z suggested that central adiposity and hypertension were the two main components correlated with cardiovascular fitness in these children. Therefore, for the outcome of fitness level, cmetS-PCA appeared to be a superior predictor to cmetS-Z.

Dysregulation of adipocytokines from adypocytes has been a subject of extensive study, which is believed to be a key mechanism in obesity-related sequelae [34]. Among the adipokines, adiponectin and leptin are the most studied. Adiponectin is anti-inflammatory, and is inversely associated with obesity and other parameters of MetS [35]; leptin, on the other hand, is positively correlated with obesity, playing a key role in regulating body mass [36]. Using a randomly selected cohort from the entire study population, we tested the association of adipocytokines with metabolic risks, as represented by cmetS. A subset of biomarkers were selected for significant association with cmetS, including adiponectin, leptin, and other inflammatory markers such as insulin and CRP. When this subset was used to model cmetS in subgroups stratified by body weight categories, a remarkable contrast was observed. In the normal/underweight group, none of the above biomarkers was significant predictor for cmetS except for adiponectin, whereas in the overweight/obese group most of these biomarkers became highly significantly predictors (Table 5). The only exception was leptin, despite that it was a significant predictor for cmetS-PCA when using the entire cohort. This result suggested that as a consequence of obesity, an entirely different relationship has emerged between metabolic risk and adipocytokines, with body fat playing a pivotal role. It has been shown by others that adiponectin is positively correlated with plasma HDLC level [37]. This is consistent with our finding that in the normal/underweight group salivary adiponectin was significantly associated only with cmetS-Z, but not with cmetS-PCA, which had much smaller loading on HDLC than the former. Incidentally, the fact that adiponectin was already inversely associated cmetS-Z in the normal group agreed well with the conclusion that a decrease in adiponectin is an early predictor of MetS in children [38]. Notably, the observation of an altered relationship between cmetS and salivary adipocytokines only in high body weight categories confirmed that these scores were good indicators for the severity of metabolic risk in the entire study population.

Due to the low prevalence of MetS in children, it is a challenge to conduct association studies using the binary variable (defined as presence of > = 3 risk factors). Therefore, the continuous score of cMets has its advantage as a more sensitive measure. In modeling fitness using the binary variable, we were unable to detect a significant effect of MetS (S1 Table) in either gender group, while a very strong effect was detected in both groups using cmetS-PCA (Table 5). Similarly, for selection of adipocytokines associated with MetS, the low prevalence made it impossible to model the binary variable.

Based on our analysis in the entire population, salivary measures appeared to be reasonable surrogates of plasma measures for evaluating metabolic risk in children. Moreover, our finding with the random cohort concurred with other findings on the relationship of blood adipocytokines and inflammatory markers with Mets in obese children [39], validating our cmetS derived from salivary parameters. We noticed, however, that in a recent study on Thai adult male, salivary adiponectin and leptin did not correlate with MetS [40]. This disagreement could be due to a number of reasons, including the difference in the way MetS was defined (continuous scores vs. binary variable), and limited power in their study rendered by a relatively small sample size.

One of the strengths of this study is the unusually large sample size, combined with homogeneity of the study subjects. As cmetS thus constructed is sample specific, which provides a relative measure of risk with each individual’s risk being compared to the study population, a large and relatively homogenous sample is advantageous. The two summary measures developed within this population enabled us to compare these two strategies comprehensively, and to our knowledge, no previous investigation has done so in children. Our data showed that these two differently constructed scores both constitute viable measures of metabolic risk in our study population. The performance of these two scores, however, was not always comparable. For instance, cmetS-PCA was a much more robust predictor for fitness level than cmetS-Z. Hence the choice of which cmetS score to use will depend on the objective of the study. Evidently, as compared to cmetS-Z, cmetS-PCA that weighs the inter-correlated components based on their variance structure would be more dynamically reflective of the data. In evaluation of longitudinal data of pediatric populations, the weighting of the components in cmetS-PCA will change at each time point a new score is constructed, reflecting the changing pattern of risk factors as body grows and matures.

One limitation of this study lies in the lack of plasma measures of HDLC and glucose in the Kuwaiti children. Due to the reticence of Kuwaiti parents, blood samples from the children were not collected. Hence, we were unable to examine the correlation between plasma and salivary measures of these two parameters in this study population. Instead, a validation study to measure this correlation was carried out in a small separate group of children instead [25]. Further investigation with plasma level of HDLC and glucose would be valuable to confirm the role of these two components in the emergent stage of Mets among children.

## Conclusion

We have constructed two continuous summary scores for metabolic syndrome from a large, metabolically at-risk pediatric population, using summation of standardized Z scores and principal component analysis, with salivary measures incorporated as surrogate for plasma measures. With cmetS as an indicator of metabolic risk, our findings demonstrated that fitness level in these children are inversely associated with MetS profile, and the relationships between Mets status and salivary adipocytokines/inflammatory markers are drastically dysregulated in overweight and obese children. Either score may be useful in investigating associations between metabolic risk and various aspects of disease process, and either could potentially be utilized to track the metabolic profile from childhood into young adulthood.

## Supporting Information

S1 Appendix (DOCX)

S1 Dataset (XLS)

S1 Table (DOCX)

## Abbreviations

AUC: Area under the curve
cmetS: Continuous metabolic syndrome score
cmetS-Z: cmetS constructed using Z score summation
cmetS-PCA: cmetS constructed using principal component analysis
CVD: Cardiovascular disease
HDLC: high density lipoprotein cholesterol
MetS: Metabolic syndrome
PCA: Principal component analysis
ROC: Receiver operating characteristic
SBP: Systolic blood pressure
DBP: Diastolic blood pressure
T2D: Type 2 diabetes
WC: Waist circumference
