# Supplementary material for: Continuous Metabolic Syndrome Scores for Children Using Salivary Biomarkers
Source: PLoS One. 2015 Sep 29;10(9):e0138979. doi: 10.1371/journal.pone.0138979 (PMC4587796; doi:10.1371/journal.pone.0138979)
Supplement: S1 Appendix — (DOCX) [file pone.0138979.s001.docx]

*HDLC Assay*

Values for HDLC were determined from the same validation study published to describe the glucose assay [24]. The results of this assay are illustrated in figure S1. Linear regression of plasma against saliva analysis resulted in the equation (Figure S1A) Plasma = 13.1*Saliva +42.5 with r=0.30 and p=0.05. In a 2x2 analysis of data (Figure S1B), 7 of the 8 subjects (88%) identified as having low HDLC (≤ 40 mg/dL) based on the commercial laboratory determination of plasma values [Quest Diagnostics, Boston MA] were identified by a saliva HDLC value of 0.6 mg/dL. We used this predictor variable to identify subjects likely to have low HDLC using a saliva measurement.

Figure S1. Analysis of HDLC values obtained from a validation study [24] in which plasma values were determined by a commercial laboratory and saliva values were determined by fluorescent assay.

Similar results have been reported by Singh et al. with a greater correlation between plasma and saliva values (r=0.57, p<0.00001)[18].

*Salivary HDL-C analysis*: The measurement of salivary HDL-C was by fluorescent assay (BioVision, Mountain View, CA, HDL and LDL/VLDD Cholesterol Quantification Kit, #K613-100) implemented on a Tecan EVO 150. In this assay, samples were centrifuged (2,800 rpm for 20 min.) following addition of precipitation buffer to separate HDL from LDL/VLDL. Cholesterol esterase hydrolyzed cholesteryl ester into free cholesterol and cholesterol oxidase specifically recognizes free cholesterol and produce products to react with probe to generate color(λ = 570 nm) and fluorescence (Ex/Em =538/587 nm).
