# Supplementary material for: Continuous Metabolic Syndrome Scores for Children Using Salivary Biomarkers
Source: PLoS One. 2015 Sep 29;10(9):e0138979. doi: 10.1371/journal.pone.0138979 (PMC4587796; doi:10.1371/journal.pone.0138979)
Supplement: S1 Table — (DOCX) [file pone.0138979.s003.docx]

Association of fitness level with binary variable of MetS (presence of >=3 risk factors)

|  | **Fitness vs. binary MetS** | |
| --- | --- | --- |
|  | **Estimate (95% CI)** | ***p* value** |
| **Boys (n=3045)** | | |
| Age (per year) | 1.56 (0.05 -3.07) | 0.04 |
| MetS (yes vs. no) | -0.34 (-10.58 – 9.91) | 0.95 |
| BMI(per unit) | 0.92 (0.71 - 1.13) | <0.0001 |
| Sleep (per hr) | 0.42 (-0.22 - 1.06) | 0.20 |
| Region |  | 0.01* |
| **Girls (n=5067)** | | |
| Age (per year) | -0.44 (-1.62 - 0.74) | 0.47 |
| MetS (yes vs. no) | 1.15 (-5.75 – 8.06) | 0.74 |
| BMI(per unit) | 0.95 (0.82 – 1.08) | <0.0001 |
| Sleep (per hr) | 0.34 ((-0.12 – 0.80) | 0.15 |
| Region |  | <0.0001* |

* indicates *p* values of Wald test for all categories in type 3 analysis
